# Differential gene expression at different stages of mesocarp development in high- and low-yielding oil palm

**DOI:** 10.1186/s12864-017-3855-7

**Published:** 2017-06-21

**Authors:** Yick Ching Wong, Huey Fang Teh, Katharina Mebus, Tony Eng Keong Ooi, Qi Bin Kwong, Ka Loo Koo, Chuang Kee Ong, Sean Mayes, Fook Tim Chew, David R. Appleton, Harikrishna Kulaveerasingam

**Affiliations:** 1 Sime Darby Technology Centre Sdn Bhd, 1st Floor, Block B, UPM-MTDC Technology Centre III, Universiti Putra Malaysia, 43400 Serdang, Selangor Malaysia; 2 Centre for Research in Biotechnology for Agriculture (CEBAR), Level 3, Institute of Research Management & Monitoring (IPPP), Research Management & Innovation Comlex, University of Malaya, 50603 Kuala Lumpur, Malaysia; 3 European Bioinformatics Institute, Welcome Trust Genome Campus, Hinxton, Cambridgeshire, CB10 1SD UK; 4 Plant and Crop Sciences, Biosciences, University of Nottingham, Sutton Bonington Campus, Loughborough, Leicestershire, LE12 5RD UK; 5 Department of Biological Sciences, Faculty of Science, National University of Singapore, off Lower Kent Ridge Road, Singapore, 117543 Singapore

**Keywords:** Oil palm, *Elaeis guineensis*, Gene expression, Expression microarray, Oil yield

## Abstract

**Background:**

The oil yield trait of oil palm is expected to involve multiple genes, environmental influences and interactions. Many of the underlying mechanisms that contribute to oil yield are still poorly understood. In this study, we used a microarray approach to study the gene expression profiles of mesocarp tissue at different developmental stages, comparing genetically related high- and low- oil yielding palms to identify genes that contributed to the higher oil-yielding palm and might contribute to the wider genetic improvement of oil palm breeding populations.

**Results:**

A total of 3412 (2001 annotated) gene candidates were found to be significantly differentially expressed between high- and low-yielding palms at at least one of the different stages of mesocarp development evaluated. Gene Ontologies (GO) enrichment analysis identified 28 significantly enriched GO terms, including regulation of transcription, fatty acid biosynthesis and metabolic processes. These differentially expressed genes comprise several transcription factors, such as, bHLH, Dof zinc finger proteins and MADS box proteins. Several genes involved in glycolysis, TCA, and fatty acid biosynthesis pathways were also found up-regulated in high-yielding oil palm, among them; pyruvate dehydrogenase E1 component Subunit Beta (PDH), ATP-citrate lyase, β- ketoacyl-ACP synthases I (KAS I), β- ketoacyl-ACP synthases III (KAS III) and ketoacyl-ACP reductase (KAR). Sucrose metabolism-related genes such as Invertase, Sucrose Synthase 2 and Sucrose Phosphatase 2 were found to be down-regulated in high-yielding oil palms, compared to the lower yield palms.

**Conclusions:**

Our findings indicate that a higher carbon flux (channeled through down-regulation of the Sucrose Synthase 2 pathway) was being utilized by up-regulated genes involved in glycolysis, TCA and fatty acid biosynthesis leading to enhanced oil production in the high-yielding oil palm. These findings are an important stepping stone to understand the processes that lead to production of high-yielding oil palms and have implications for breeding to maximize oil production.

**Electronic supplementary material:**

The online version of this article (doi:10.1186/s12864-017-3855-7) contains supplementary material, which is available to authorized users.

## Background

Oil palm (*Elaeis guineensis* Jacq.) is the most productive vegetable oil crop in the world with each hectare of oil palm plantation producing up to ten times more oil per hectare per year than other oilseeds or other oil crops [1–3]. Moreover, global palm oil production accounted for 63% of all edible oils, with Indonesia the main contributor to world palm oil production, followed by Malaysia in 2014/15 [4]. With an increasing world population and global food demand, oil palm breeding programs aim to improve oil yield per hectare per year as a necessary component of achieving food security in a sustainable manner [5].

Interestingly, while oil palm, sago palm (*Cycas revoluta*), sugar palm *(Arenga pinnata))*, date palm (*Phoenix dactylifera)*, and coconut palm (*Cocos nucifera)* all belong to the same palm family, it is only the fruits of the oil palm that produce oil, while its close relatives all produce sugar or starch as their major storage compound [6]. This fascinating fact has spurred a number of studies aimed at elucidating the mechanisms of oil biosynthesis and the specific molecular pathways in oil palm that differ from those in its carbohydrate producing relatives. Bourgis et al. compared the transcriptome and metabolites of oil palm and date palm mesocarp to investigate the mechanistic differences that lead to oil and sugar accumulation, respectively [7]. They found that the high oil content in oil palm mesocarp is associated with higher transcript levels of enzymes involved in fatty acid biosynthesis, as might be expected, as well as key enzymes from the plastidial carbon metabolism, compared to the gene expression in developing fruits of date palm. Studying regulatory mechanisms at various developmental stages of oil palm mesocarp by taking a 454 pyrosequencing-derived transcriptome approach, Tranbarger et al. [8] identified the transcription factor WRINKLED1, which coordinates several genes involved in fatty acid synthesis, as an important oil biosynthesis regulator. Furthermore, the comparative transcriptome analysis of three different tissues (endosperm, embryo and mesocarp) of oil palm by Dussert et al. [9] provided insights into the controlling mechanisms responsible for the variation of the fatty acid composition observed in these tissues. For example, lauric acid predominates in the endosperm, while palmitic and oleic acid are the main components of oil found in the mesocarp. This is of great interest to the palm oil industry as different types of fatty acid have their own applications. For example, lauric acid is widely used in soap and the cosmetic industry, while mesocarp oil rich in oleic acid is preferable for cooking purposes.

A number of studies have investigated the mechanisms of oil biosynthesis pathways between high and low oil-yielding lines in other oil producing crops such as rapeseed [10–13], soybean [14], maize [15, 16] and sunflower [17]. These studies took a variety of approaches, including microarray/gene expression [10, 11, 14], proteomics studies [15, 18], microscopic analysis of oil bodies [13] and enzyme activities [17, 19] to try to elucidate the gene functions which might contribute to comparatively high or low oil content in these oil crops.

Previously, we have reported differences in metabolite profiles at six developmental points in high-yielding oil palm mesocarp compared to low-yielding palms [20]. The palms analysed were derived from the same genetic background and in the same planting location to minimize (but not eliminate) genetic and environmental factors. We found that the glycolysis-associated metabolites, nucleosides and amino acids exhibited complex differential patterns between high and low-yielding palms. Furthermore, proteomic analysis of the same samples suggested that enzymes involved in oxidative phosphorylation were crucial for oil biosynthesis in high yielding oil palm [21]. In addition, the expression level of the β subunit of the ATP synthase complex was found to be higher in high-yielding palms than that in low-yielding palms during the maturation stage. From both metabolomic and proteomic studies, the results suggested that an increased energy supply was critical for greater oil production in high-yielding oil palms.

To better understand the oil yield drivers at the genetic level, we here used a custom expression microarray to analyse gene expression in the same sets of palm, to identify genes that were differentially expressed between the high- and low-yielding oil palms.

## Methods

### Plant material

All oil palm mesocarp tissues were obtained from palms in field trials located in the Sime Darby Plantation at Carey Island, Selangor, Malaysia. A total of eight palms with similar genetic backgrounds (Serdang Avenue *dura* x AVROS *pisifera*) were selected for each of the high-yielding (HY) and low-yielding (LY) classification groups based on the average yearly production of oil yield (oil/ha/year) as assessed by bunch analysis over at least 5 years of recording. The selected palms from the high- and low-yielding groups yielded an average of 10.7 and 5.5 tons of oil per hectare per year, respectively, over the breeding trial [20, 22]. Six inflorescences were open pollinated and fruit bunches were harvested at different time points, namely, 12, 14, 16, 18, 20 and 22 weeks after pollination (WAP), for each of the 16 palms in the experiment. The oil palm fruits from each bunch collected at each sampling were randomized. The fresh mesocarp tissue was then collected from randomized fruits and flash frozen in liquid nitrogen before storing at -80 °C.

### Oil palm mesocarp RNA extraction

Total RNA was extracted from oil palm mesocarp tissue using the RNA extraction method described in Wong et al. [23]. Concentration and purity of total RNA was determined by Nanodrop spectrophotometer quantification. The AU 260/280 and AU 260/230 were measured and samples with ratios of 1.8-2.0 were accepted. Gel electrophoresis was performed on one microgram of total RNA by separation on a 1% agarose gel in TAE buffer to further determine the RNA quality. Gels were imaged and documented using an AlphaImager 2200 from Alpha Innotech. The quality of total RNA was further analyzed using an Agilent Bioanalyzer (G2938C). Samples with 28S to 18S ratios of greater than 2:1 and RIN (RNA Integrity Number) score greater than 7 were selected for microarray work.

### Custom Design of oil Palm Mesocarp Array

The custom gene expression array for oil palm mesocarp was based on the Agilent microarray platform in 2 × 105 K format. The probes were designed based on 31,794 sequences from mesocarp transcriptome sequencing derived from the six different stages of mesocarp development, with annotation obtained by comparison to the Uniprot database, as has been described in a previous study [23]. The transcriptome sequences have been deposited in the European Nucleotide Archive (ENA) (accession numbers: LM611910 to LM643713). Probes were designed using Agilent’s internal design program through the “eArray” website. Each of the transcriptome sequences was represented by three individual probes [23].

### Synthesis of cRNA, microarray hybridization and scanning

Total RNA samples from mesocarp were labeled with a one-color Cy3 dye according to the Low Input Quick Amp Labeling protocol (version 6.0, December 2009) provided by Agilent [24]. A total of 100 ng of total RNA was used to synthesize cRNA using Oligo dT-Promoter primer (provided inside the kit) and labeled with Cy3 dye. Labeled cRNAs were hybridized onto the mesocarp array at 65 °C for 16 h. After hybridization, the mesocarp array was washed twice with wash buffer 1 at room temperature for 1 min and wash buffer 2 for 1 min at 37 °C. The array was then air-dried for a few seconds and scanned using an Agilent microarray scanner (SG11350602) [23].

### Data extraction, analysis, selection of candidates and classification

Raw microarray data was extracted from scanned images using Agilent’s Feature Extraction software (version 10.7.31). After extraction, raw microarray data was normalized with the Quantile normalization algorithm in the R package, limma (Linear Models for Microarray Data, version 2.13.1) [25]. After normalization, gene probe signal values from high-yielding (HY) group samples were compared to probe signal values of samples from the low-yielding (LY) group. A t-test determined the statistical significance in the differences between the two populations. The differentially expressed probes were further filtered based on *p*-values. The expression signal difference threshold in the experiment was set at 1.5 fold change (0.6 Log_2_ fold change) and *p*-value threshold set at <0.05 [26]. The differentially expressed candidates from each time point were annotated using the Gene Ontology (GO) categories of the Blast2GO program to reflect their likely gene functions. The program was used to obtain Gene Ontology (GO) annotation for all candidates, which were further classified according to GO using the CateGOrizer web tool [27]. To identify significantly enriched GO terms, enrichment analysis was performed on all differentially expressed genes with GO annotation using a chi-square test. The significance threshold for enriched GO was set at a *p*-value <0.05.

### Quantitative real-time PCR

The microarray expression data was validated using quantitative real-time PCR (qPCR). Two micrograms of total RNA from each sample were subjected to a reverse transcription reaction using Omniscript Reverse Transcriptase with standard conditions as recommended by the manufacturer (QIAGEN). The first strand cDNA was synthesized using a random hexamer primers. Specific primers were designed using the Primer Premier 5.0 software. The qPCR reaction mix and cycling conditions were based on the optimized conditions suggested by BIO-RAD of 95 °C (1 min) for 1 cycle and followed by 95 °C (15 s) and 55 °C (35 s) for 40 cycles. Relative expression of each transcript was analyzed using the qBase Plus 2.0 software [28] and normalized against two reference genes, Cyp2 and GRAS, as previously reported [23, 29].

## Results and discussion

### Functional classification and comparison of differentially expressed candidate genes during mesocarp development in high- and low-yielding oil palm

A total of 3412 non-redundant differentially expressed isotigs (annotated and unannotated) were obtained with the cut-off fold-change set at 0.6 (Log_2_ fold change) and *p*-value at <0.05 (Additional file 1). Out of the 3412 isotigs, 2001 isotigs were annotated and 1411 were not annotated based on the Uniprot database. Due to sampling palms from the open field and the lack of an applied treatment beyond the natural oil production differences observed, all differentially expressed candidates became statistically insignificant for all the time points analyzed when False Discovery Rate (FDR) was applied (8 samples of HY, 8 samples from LY for 12-20 WAP, 6 samples from HY and 7 samples from LY for 22 WAP). This lack of significance may be due to the samples being natural variants for oil yield rather than samples responding to treatment. Similar selection criteria were also used in the study by Chen et al. [26]. However, to avoid false positive candidates due to the lower stringency used, selected candidates with differential expression between the populations were further validated by qPCR before further functional analysis (Additional file 2).

**Fig. 1:**
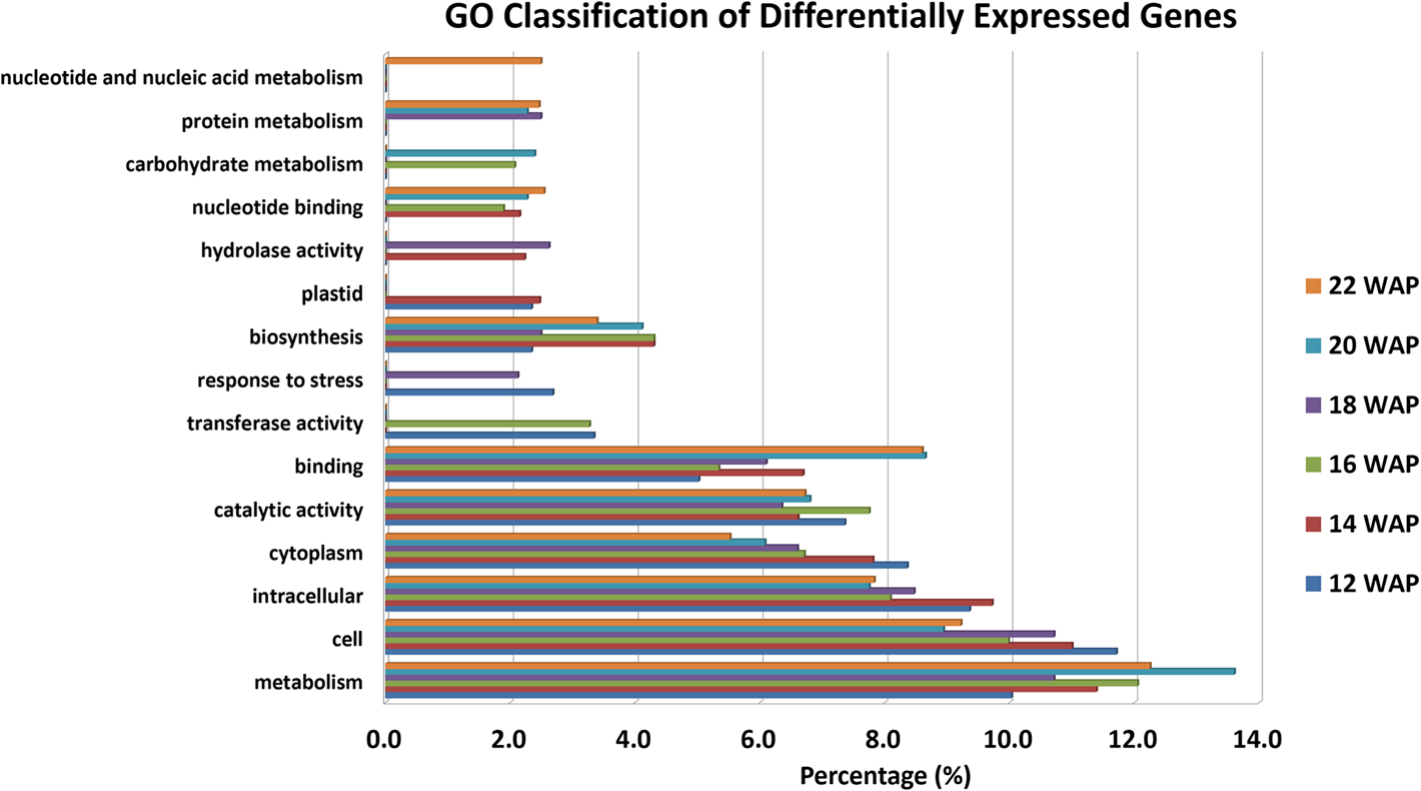
GO classification comparison across six different time points for differentially expressed genes. The ten highest representative GO categories from each time point are shown in the bar chart. The total of these ten GO categories represents over 55% of GO counts in each time point

All the differentially expressed genes were classified in accordance with the GO terminology determined using cateGOrizer. More than 55% of the differentially expressed genes were represented in the top ten GO classifications of each time point. Most of the differentially expressed genes were classified under metabolism (GO:0008152) and cell (GO:0005623), suggesting that the metabolic processes could be the key factor differentiating between HY and LY oil palm (Fig. 1), as might be expected. To identify significantly enriched GO terms, all differentially expressed genes with GO were further tested using a chi-square test (Additional file 3).

We identified in total 28 significantly enriched GO terms in biological processes (12), cell components (7) and molecular functions (9) at different time points in this study (Table 1). According to the biological process GO categories (Table 1), the GO terms related to regulation of transcription (GO:0006278), translation (GO:0006412) and various metabolic and biosynthetic processes (GO:0006206, GO:0006278, GO:0006633 and GO:0008152) were also enriched. The candidates from the metabolic process category (GO:0008152 and GO:0006633) included genes involved in fatty acid biosynthesis and metabolic processes, such as 3-oxoacyl- acyl-carrier-protein synthase I (KAS I), 3-oxoacyl-acyl-carrier-protein synthase III (KAS III) and 3-oxoacyl-acyl-carrier-protein reductase (KAR). Not surprisingly, fatty acid biosynthesis genes are critical and have been identified as a major contributor to the differences seen during fruit ripening when comparing oil and date palm fruit [7]. The highest numbers (5) of enriched GO terms for biological processes were found at 22 WAP. These included pyrimidine nucleobase metabolic processes (GO:0006206), RNA-dependent DNA biosynthetic processes (GO:0006278), regulation of transcription (GO:0006355), DNA integration (GO:0015074) and photosynthesis (GO:0015979). Most of the genes found in these GO were down-regulated in HY samples. They included many transcription factors such as Agamous-like MADS-box protein, MADS-boxtranscriptionfactor, GATA transcription factor, Homeobox-leucine zipper protein and zinc finger protein.

**Table 1:**
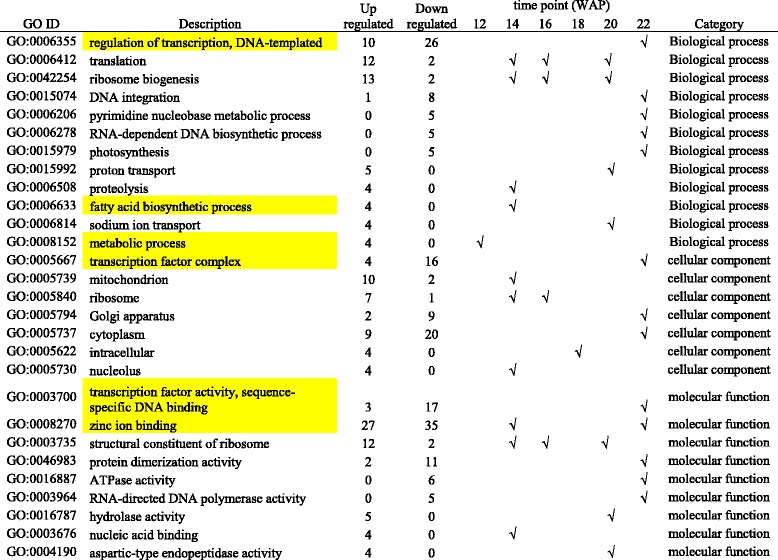
Gene ontology enrichment summary of differentially expressed genes (*p*-value <0.05)

Highlighted GOs consist candidates genes related to regulation of transcription (transcription factors), glycolysis, TCA and oil biosynthesis pathways

For cellular components, the significant enriched GO terms included ribosome (GO:0005840, 12 WAP and 14 WAP), nucleolus (GO:0005730), cytoplasm (GO:0005737), mitochondrion (GO:0005739), Golgi apparatus (GO:0005794) and transcription factor complex (GO:0005667). In the molecular function category, three enriched GO terms were found to be related to binding activities such as nucleic acid binding (GO:0003676), transcription factor activity, sequence-specific DNA binding (GO:0003700) and zinc ion binding (GO:0008270). Further, we found differentially expressed genes in the TCA cycle for the Succinate Dehydrogenase and Pyruvate Dehydrogenase E1 components of the glycolysis pathway under the zinc ion binding category (GO:0008270).

Overall, most of the enriched GO terms were involved in general metabolism such as ribosome biogenesis, translation, structural constituents of the ribosome and hydrolase activity, as would be expected to be up-regulated in HY oil palms compared to LY palms. Those enriched GOs of particular interest to us, such as regulation of transcription, metabolic process, fatty acid biosynthetic process, transcription factor activity and zinc ion binding will be discussed further in the following sections. The candidate genes classified under these GO terms are likely to be involved directly or indirectly in oil production and accumulation in HY oil palms.

### Differentially expressed transcription factors (TFs) between high- and low-yielding oil palm mesocarp

In this study, enriched transcription factors (TFs) were found in the regulation of transcription (GO:0006355) in biological processes; the transcription factor complex (GO:0005667) in cellular components; transcription factor activity; and sequence-specific DNA binding (GO:0003700) in molecular functions.

These TFs were differentially expressed at both the early and late stages of oil palm mesocarp development and ripening. Differentially expressed TFs between HY and LY palms are shown in Table 2, for example, MYB, AP2/ERF, MADS, GATA, Dof (DNA-binding with one finger), bHLH (Basic Helix loop helix), and NAC (Table 2). Results from Liu et al. showed that 42.9% of transcription factors that were differentially expressed in *Arabidopsis* between transgenic lines (>40% higher in seed oil) and wild-type belonged to five main families, namely: the WRKY domain family, the MYB domain and related transcription factor family, the basic helix-loop-helix (bHLH family), the AP2/EREBP family, and the C2H2 zinc finger family [30]. Niu et al. found approximately 320 transcription factors to be expressed during *Brassica napus* seed development, but only seven had an expression pattern similar to the FA synthesis-related genes including AP2, and MADS family members, bHLH, and a zinc finger TF [31]. A few transcription factors have been reported to regulate FA metabolism related to intracellular signaling and storage. Comparative transcriptomic analysis of two *Brassica napus* near-isogenic lines (high oil content vs. low oil content) by Wang et al., identified TFs of MYB, AP2/ERF and Dof which were found to be differentially expressed [32].

**Table 2:** Differentially expressed transcription factors compared between HY and LY oil palm

Isotig	Gene Coded	Fold Change (log_2_)	*p*-value	Differentially expressed time point (WAP)
34,113	MYB21	0.744	0.007	22
47,235	MYB44	0.7342	0	22
18,202	GATA28	0.7456	0.006	22
18,620	GATA10	1.1379	0.003	22
18,471	APETALA2	0.6996	0.035	20
27,438	MADS34	0.979	0	20
31,823	MADS2	−1.0955	0.004	22
5007	MADS21	−0.8081	0.004	22
0231	Agamous-like MADS-box	−0.6537	0.024	22
14,030	NAC domain-containing protein 90	0.8877	0.00047	22
19,662	NAC domain-containing protein 48	0.6948	0.0114	22
26,756	Dof zinc finger protein 5.2	−0.6921	0.0122	22
34,017	bHLH113	0.7734	0.00231	14
19,157	bHLH47	0.6275	0.01199	14
28,709	bHLH93	0.9264	0.00237	20
17,360	bHLH62	0.9195	0.02858	22
37,216	bHLH122	1.1763	0.01407	22

In our study, two MYB transcription factors, namely MYB21 and MYB44, were up-regulated in HY palms at 22 WAP. During ripening, species such as oil palm and olive accumulate anthocyanin in their fruits. MYB has also been found in abundance in olive and was also found to be directly correlated to anthocyanin accumulation in olive fruit [33], suggesting a relationship between MYB and anthocyanin accumulation during the ripening stage in oil palm fruit development.

Several bHLH transcription factors were found to be differentially expressed at various time points in our study (14 WAP, 20 WAP and 22 WAP). This family of TFs regulates growth, development, and stress responses in plants. Liu et al. demonstrated that the bHLH (*SPATULA*) TF regulates seed size and seed fatty acid content in *Arabidopsis* [34], suggesting that *SPATULA* promotes fatty acid accumulation through inhibition of seed storage protein-associated genes and the induction of fatty acid associated genes during seed development [34]. Two bHLH TFs (bHLH47 & bHLH113) were found up-regulated at 14 WAP in our study at the stage before oil biosynthesis starts. These results indicate these bHLH TFs to be having a similar function to that suggested by Liu et al. which is to promote fatty acid biosynthesis associated genes to produce higher oil yield in HY oil palm.

Transcripts coding for APETALA 2, MADS 34, MADS 2, and MADS 21 whose homologues are known to play a role in fruit ripening were also found to be differentially expressed in our study. APETALA 2 and MADS 34 were up-regulated in high yielding palms at 20 WAP, while MADS 2 and MADS 21 were down-regulated at 22 WAP. WRI1 and APETALA (AP2) are AP2/EREBP domain-containing TFs. WRI1 regulates glycolysis and oil biosynthesis pathways in oil seeds [35]. In a recent study by Jing et al., WRI transcription factors genes were found to be up-regulated more in the mesocarp of *tenera* palms than in its parental *dura* and *pisifera* palms [36]. However, in our study, WRI1 did not display a significant differential of expression between HY and LY palms. On the other hand, AP2, the other TF containing the AP2/EREBP domain, was found to be up-regulated in week 20 WAP in HY palms compared to LY palms. AP2 has been found to be significant in embryo, endosperm and seed coat development in *Arabidopsis* and can influence seed size [37]. In *Arabidopsis*, AP2 mutants produce larger seeds, and their mature embryos display more and larger cells than the wild type [38, 39]. In turn, this also suggests that AP2 normally suppresses the growth of the endosperm by controlling the size of the vacuole [37]. AP2 also have been shown to affect carotenoid biosynthesis and accumulation through the regulation of fruit ripening [40], which would coincide with the up-regulation of the AP2 in HY oil palm at a later stage of fruit development found in our study.

MADS box proteins are known to be key regulators of fruit ripening [41–43]. In tomato, the Ripening Inhibitor (RIN) gene encodes a putative MADS-box transcription factor that controls tomato fruit ripening, and the ripening inhibitor (rin) mutation yields a non-ripening fruit [42]. In strawberry, a SEP1/2-like (FaMADS9) mutation leads to the inhibition of normal development and ripening [43]. Gene silencing of the SQUAMOSA-class MADS-box TF, VmTDR4, has been shown to result in a reduction of anthocyanin levels in fully ripe fruit through the direct or indirect control of transcription factors belonging to the R2R3 MYB family [41]. The time points with differential expression (20 WAP and 22 WAP) of the putative oil palm MADS proteins in the microarray data coincides with ethylene production of oil palm fruit during maturation, suggesting that the putative oil palm MADS proteins may regulate genes in the ethylene production pathway. Elitzue et al. also suggested *MaMADS2* to act in the banana pulp upstream of the increase in ethylene production [44]. These genes may play a regulatory role in the ripening process by stimulating the ripening process in HY palms, resulting in earlier and faster lipid accumulation. Parra et al.’s comparative transcriptional profiling analysis of olive ripe-fruit pericarp and abscission zone tissues [45] found TF, such as MADS-box TF, MYB, NAC, bZIP, that were up-regulated in ripe olive fruit at the late stage of ripening. They postulated TFs from these families have potentially important roles in mediating late events during olive fruit ripening.

The Dof zinc finger protein 5 was found to be down-regulated in 22 WAP in our study. Dof has been found associated with seed storage protein (SSP) genes. The AACA motif was repeatedly found in SSP gene promoters, bound by TFs of the bZIP, Dof proteins and MYB domains [46]. Kana et al. [47] demonstrated that the oil/protein ratio in *Arabidopsis* seeds was determined by sequential activation of genes in oil biosynthesis followed by protein synthesis. Through mutational knockout of a major seed storage protein, it was possible to enhance seed oil by 170% compared to the wild-type [47]. The down-regulation of the Dof protein-encoding gene in the HY oil palm compared to the LY palms could indicate that protein synthesis at the later stages of oil palm mesocarp development was suppressed in favour of higher oil synthesis. However, the actual mechanism of TF involvement is not yet fully understood and remains to be investigated further.

### Differentially expressed genes related to fatty acid biosynthesis

**Fig. 2:**
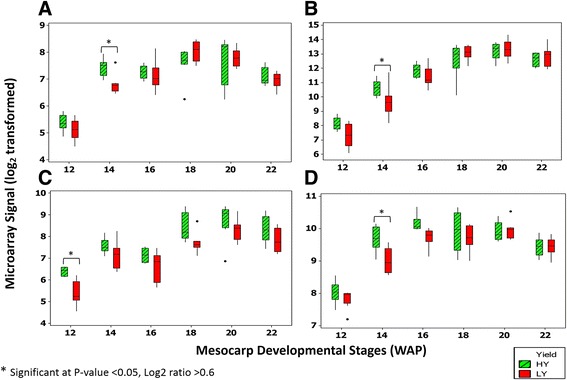
Differentially expressed genes involved in fatty acid biosynthesis pathway. **a** KAR (ketoacyl-ACP reductase), **b** KAS III (β- ketoacyl-ACP synthases III), **c** KAS I (β- ketoacyl-ACP synthases I) (Isotig17406), **d** KAS I (Isotig26074). Eight biological replicates of HY and LY were used for each time point (12-20 WAP) except for 22 WAP (six HY and seven LY)

The plastidial fatty acid synthesis pathway is the key pathway in oil-producing plants. Several genes from this pathway were identified as being differentially expressed between HY and LY palms. These genes were classified in fatty acid biosynthetic processes (GO:0006633) and metabolic processes (GO:0008152) in the GO classification. Interestingly, both GO terms were significantly enriched in this study. Among these genes were β- ketoacyl-ACP synthases I (KAS I), β- ketoacyl-ACP synthases III (KAS III) and ketoacyl-ACP reductase (KAR) (Fig. 2). Another gene that encodes trans-2-enoyl-CoA reductase was also found to be enriched (GO:0008270) at 14 WAP in HY compared to LY palms. Notably, most of these genes were categorized in the fatty acid synthesis sub-category, which has been described by Bourgis et al. as the sub-category displaying major differences between oil palm and date palm during fruit ripening [7]. We also identified Acetyl-coenzyme A carboxylase carboxyl transferase subunit alpha (Isotig18065) to be up-regulated at 12 WAP in HY oil palm (Additional file 4).

ACCase is considered the first committed step in the fatty acid biosynthesis pathway. The plastidial heteromeric ACCase isozyme catalyzes the formation of malonyl-CoA to provide two carbon units for use by the Fatty Acid (FA) Synthase complex in the plastid while the cytosolic homomeric ACCase isozyme provides malonyl-CoA for further elongation of FAs in the endoplasmic reticulum (ER) [48]. ACCase consists of three nuclear encoded subunits (biotin carboxyl carrier protein, biotin carboxylase and carboxyl transferase α-subunit) and a plastid-encoded subunit (carboxyl transferase β-subunit). The plastid-encoded subunit seems to be crucial for the accumulation of heteromeric ACCase and for increasing oil content [49]. Plants also have a homomeric ACCase localized in the cytosol that does not appear to play any major role in fatty acid biosynthesis, which takes place almost exclusively in plastids. However, by targeting ACCase to plastids, oil content was increased five-fold in potato tubers [50] and 5% in rapeseed [48]. Our expression microarray data found the ACCase subunit alpha to have a higher expression in HY palms compared to LY palms at week 12 (Additional file 4). This is in accordance with observed proteomics data at week 12 and 14 in HY oil palm mesocarp samples [21]. It is also in accordance with results reported by Nakkaew et al**.** comparing ACCase expression levels between high production oil palm (measured as >150 FFB/palm) and low production oil palm (<140 FFB/palm) [51]. Using semi-quantitative RT-PCR, they demonstrated for the first time that a higher level of ACCase expression is correlated with higher productivity and oil content in the oil palm material examined. They further validated the data with real-time PCR analysis confirming a direct correlation of ACCase expression levels with oil palm productivity for this material.

We found two transcripts (isotig17406 and isotig26074) that encode for KAS I (Fig. 2(c) & (d)) and one transcript coding for KAS III (Fig. 2(b)) to be differentially expressed between HY and LY oil palm at 12 and 14 WAP. KAS I is essential for normal seed oil accumulation in *Arabidopsis.* KAS I deficiency in *Arabidopsis* mutants results in a reduction of fatty acid levels (~33.6% of the wild type) and also a disruption of embryo development before the globular stage [52]. KAS III has been shown to catalyze the first elongation reaction of type II fatty acid synthesis in plant plastids. The cycle of elongation starts with the condensation of malonyl-ACP with Acetyl-CoA to produce acetoacetyl-ACP which is further reduced by KAR to yield the β-hydroxyacyl derivative. KAS III and KAR were found to be up-regulated in hickory (*Carya cathayensis* Sarg.) at the late cotyledon stage, which demonstrated that the fatty acids were more rapidly synthesized and their corresponding lipids instantly transferred to oil bodies for energy reserve [53].

### Differentially expressed genes related to the Glycolytic and TCA pathways

**Fig. 3:**
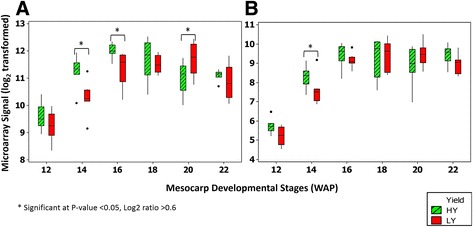
Differentially expressed genes in the glycolysis pathway (**a**) GAPDH (Glyceraldehyde-3-phosphate dehydrogenase) and (**b**) Pyruvate Dehydrogenase (E1 component). Eight biological replicates of HY and LY were used for each time point (12-20 WAP) except for 22 WAP (six HY and seven LY)

Another group of GO terms, zinc ion binding (GO:0008270), was found to be enriched in the analysis. Interestingly, several genes classified in this GO are involved in the glycolysis and TCA pathways, such as Glyceraldehyde-3-phosphate dehydrogenase (GAPDH), Pyruvate Dehydrogenase E1 Component Subunit Beta (PDH) and Succinate Dehydrogenase. Glycolysis is the central pathway of carbon metabolism as it converts sugars into precursors for protein and fatty acid synthesis while concomitantly producing ATP by substrate level phosphorylation. The glycolytic pathway is the principal source of carbon skeletons and reducing power for lipid biosynthesis. In our previous study, we found that the intermediate metabolites involved in glycolysis exhibited varied trends between the HY and LY oil palm groups during the fruit developmental stages. The concentrations and trends of early intermediates (glucose, glucose-1-phophate and glucose-6-phosphate) were similar across HY and LY, while fructose-6-phosphate appeared at significantly higher concentrations in HY during lipid biosynthesis [20]. Levels of fructose-1,6-biphosphate appeared to be lower in HY, preceding the start of lipid biosynthesis, but increase during maturation and finally exceed the levels measured in LY samples at 20 WAP [20]. In this study, GAPDH was found to be up-regulated in HY oil palm at 14 and 16 WAP (Fig. 3(a)) compared to the LY palms at the same stages. GAPDH is involved in glycolysis and catalyzes the breakdown of glucose for energy and carbon molecules. A study has shown that GAPDH levels were directly correlated with seed oil accumulation with a greater than 3% increase in seed oil in a seed-specific overexpression line in *Arabidopsis* [54].

Pyruvate dehydrogenase E1 Component Subunit Beta (PDH) was up-regulated in HY palms at week 14 WAP (Fig. 3(b)) compared to LY at the same stage. Pyruvate is the direct precursor of carbon for fatty acid biosynthesis in most oil-synthesizing tissues, and a plastidial PDH supplies acetyl-CoA for ACCase to be converted to malonyl-CoA through carboxylation. Transcriptional profiling in oil palm identified subunits of PDH with 50-fold higher transcript levels compared to that in date palm, reinforcing transcriptional regulation as a major means of influencing FA supply for Triacylglyceride (TAG) synthesis [7] rather than carbohydrate storage. Oxidative decarboxylation of pyruvate to acetyl-CoA by PDH is also one of the important steps in fatty acid biosynthesis. PDH is a four-subunit enzyme complex of pyruvate decarboxylase (E1-α and E1-β subunits), dihydrolipoyl acetyltransferase (E2 subunit) and dihydrolipoamide dehydrogenase (E3 subunit) enzymes [55].

**Fig. 4:**
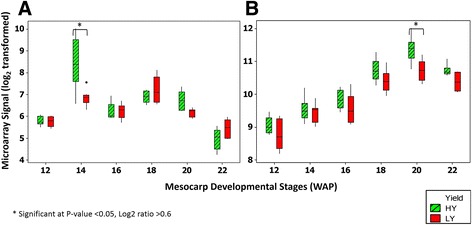
Differentially expressed genes in the TCA Cycle. (**a**) Succinate Dehydrogenase and (**b**) ATP-Citrate Lyase. Eight biological replicates of HY and LY were used for each time point (12-20 WAP) except for 22 WAP (six HY and seven LY)

The TCA cycle is the core machinery for carbon skeletons and energy. Our data indicates an up-regulation of Succinate Dehydrogenase in HY palms at 14 WAP, as shown in Fig. 4(a). Succinate Dehydrogenase plays an important role in the TCA cycle as catalyst in the oxidation of succinate to fumarate and also in the respiratory electron transport chain. It has been shown that Succinate Dehydrogenase is capable of generating reactive oxygen species, which could play an important role in growth regulation as well as stress-related gene expression in plants [56]. We also identified ATP-citrate lyase as significantly up-regulated in HY oil palm at 20 WAP (Fig. 4(b)) compared to the same stage in LY palms. ATP-citrate lyase catalyzes the conversion of citric acid into acetyl-CoA and thus represents a source of acetyl-CoA for fatty acid biosynthesis. Studies in other oil-producing crops have confirmed this link between increased ATP-citrate lyase expression and higher oil accumulation. In a transgenic *Brassica napus* line seed oil content increased upon up-regulation of ATP citrate lyase [57]. The temporal distribution of ATP citrate lyase activity in developing seeds of *Brassica napus* L. was also found to be positively correlated with acetyl-CoA carboxylase and the overall rate of lipid biosynthesis [58].

In a previous study, we found citric acid, isocitric acid and, more markedly, 2-oxoglutaric acid to all have significantly lower concentrations in the HY group of palms just preceding and during the early stages of lipid biosynthesis (14-18 WAP) [20]. Our transcriptomics data is in accordance with the metabolomics data obtained [20]. Malic acid exhibited a very different concentration profile between the HY and LY groups, maintaining a significantly higher concentration in HY palms from 12 WAP through to the mid-point of lipid biosynthesis (18 WAP) when it dropped to a level similar to that of the LY group [20]. According to Teh et al., all of the organic acids in the TCA cycle were at their lowest concentrations during the final stage of fruit maturation. A possible scenario is that in high-yielding palms the TCA cycle is up-regulated or the enzymatic shunts are up-regulated. This causes the comparatively lower concentration of input metabolites, with ATP-citrate lyase the key gene in increasing the flux through the TCA towards oil biosynthesis.

### Higher carbon flux into lipid biosynthesis in HY palms

**Fig. 5:**
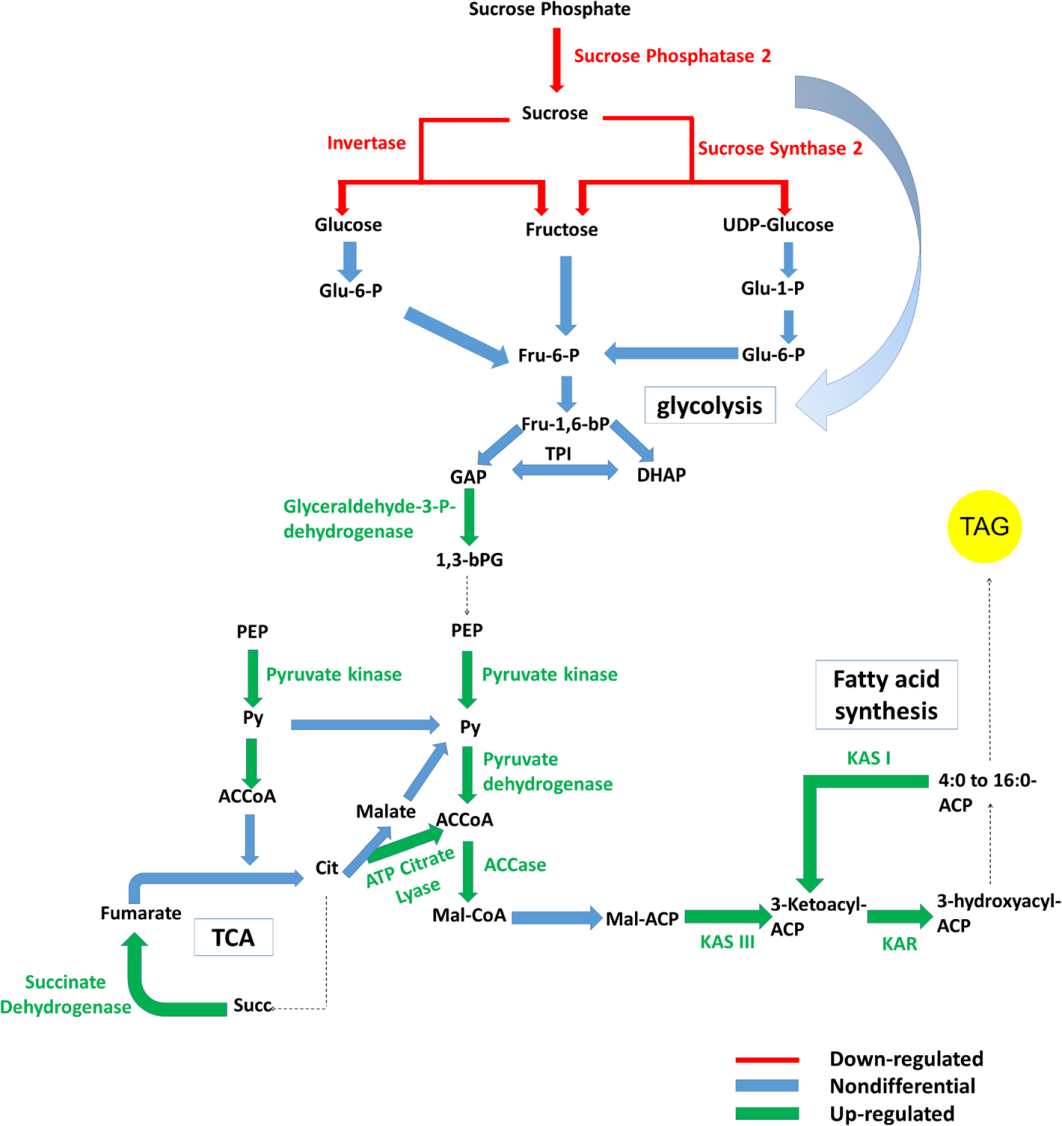
Summary of the differentially expressed genes in pathways that are involved directly or indirectly in lipid biosynthesis in high-yielding oil palm. Green represents up-regulated, red represents down-regulated genes and blue represents non-differentially expressed genes. The curved arrow represents the carbon flux that is channeled through the Sucrose Synthase 2 pathway

Lipids (TAG) assembled in the ER are formed using acetyl-CoA as the precursor from the glycolytic pathway. Further upstream of the glycolysis pathway, fructose-6-phosphate converted from sucrose phosphate is used as a carbon source to be converted into pyruvate to enter the glycolysis pathway. Five isotigs related to sucrose and starch metabolism (GO:0005982) were identified in the microarray study (Additional file 4). In our GO enrichment analysis, this set of genes categorized in starch metabolic processes were not significantly enriched due to insignificant differentiation between up- and down-regulated genes in this group. However, these genes were found to be differentially expressed at significant levels between HY and LY palms when analyzed individually. Isotigs similar to sucrose synthase 2 (SUS2) and sucrose phosphatase were expressed at lower levels in HY palms. A 4-α-glucanotransferase or DPE2-like gene was expressed at higher levels in HY than in LY palms. In addition, one β-fructofuranosidase 1-like isotig (also known as Invertase 1) appeared to be down-regulated from 12 to 18 WAP while being expressed at higher levels from 18 to 22 WAP in HY palms compared to LY palms. Interestingly, our microarray analysis found SUS2 to be expressed at higher levels than invertase throughout the ripening stages (Additional file 4). We propose that the sucrose in oil palm mesocarp is mainly hydrolysed via the sucrose synthase path and channeled into glycolysis as a carbon source for lipid biosynthesis in the mesocarp rather than being channeled through the invertase pathway (Fig. 5). Furthermore, SUS2 expression steadily increased towards the end of the maturation stage, coinciding with oil accumulation in the mesocarp. This timing suggests an important role for this gene as a sucrose-cleaving enzyme providing a carbon source for oil biosynthesis in oil palm mesocarp. Indeed, this is also suggested by a study by Niu et al. [59] showing *SuSy2* to be gradually up-regulated during the developing stages of *Lindera glauca* fruits. At maturation, SUS2 was found to be down-regulated in HY compared to LY palms at 20 WAP. This difference has also been observed in a comparison of canola lines with high and low oil content [60]. The decreased activity of SUS2 may lead to increased lipid accumulation in the mesocarp, suggesting this to be an effect of carbon channeling away from starch towards oil biosynthesis. Developing *Arabidopsis* seeds showed a similar scenario [61] where seeds from SUS2 mutants had a 55% higher lipid content than wild type at 9-15 day after flowering. Downstream genes such as pyruvate kinase, pyruvate dehydrogenase, ATP-citrate lyase and glycerol-3-phosphate acyltransferase that are involved in glycolysis, TCA and FA biosynthesis were expressed at higher levels in HY palms. These genes are required for carbohydrate metabolism and peak early in mesocarp development, followed by genes for oil synthesis in later stages. An increased proportion of the carbon channeled into fatty acid synthesis by up- or down-regulation of multiple enzyme activities (source) increase the supply of pyruvate, which leads to greater oil production through the FA biosynthesis machinery (Fig. 5). Hence, altered carbon flux towards glycolysis and TCA cycle directs a greater flux towards acetyl-CoA, a precursor for fatty acid biosynthesis leading to increased lipid accumulation.

## Conclusions

To our knowledge, this study is the first comparative analysis of global gene expression between high- and low-yielding oil palms. Our analysis identified several transcription factors including bHLH, Dof zinc finger and MADS box protein classes. Many of these transcription factors are related to lipid accumulation in other oil species and possibly in fruit ripening in oil palm, but further studies are needed to fully understand their function. Our results also showed a number of up-regulated genes involved in glycolysis, TCA and fatty acids pathways such as PDH, ATP-citrate lyase, KAS I, KAS III and KAR. The most notable finding of this study is that sucrose metabolism-related genes (Invertase 1, Sucrose Synthase 2 and Sucrose Phosphatase 2) had significantly lower expression in the HY than in the LY oil palm. These genes may either reflect differential fluxes between the pathways which lead to significantly higher oil accumulation in the HY palms compared to the LY palms or may even be some of the key drivers of higher oil production in HY oil palms. This study takes an important step in identifying the genes that regulate this differential metabolite flux and could be manipulated to enhance oil content of oil palm in future breeding programmes.

## Additional files

Additional file 1: Normalized microarray data of all differentially expressed isotigs for all biological samples at different time points. Selection criteria were set at *P* < 0.05 and 1.5 fold change (0.6 Log_2_ fold change). (XLSX 931 kb)

Additional file 2: qPCR primer efficiencies and gene expression comparison between HY and LY oil palm group at specific time points for selected differentially expressed genes. (DOCX 66 kb)

Additional file 3: GO enrichment analysis of differentially expressed genes at different time points (*P* < 0.05). Coloured GO represent enriched GO terms at specific time points. (XLSX 365 kb)

Additional file 4: Expression patterns of ACCase Subunit Alpha and genes involved in starch and sucrose metabolism pathway throughout fruit ripening in oil palm (12 – 22 WAP). (DOCX 1353 kb)

## Abbreviations

cDNA: Complementary Deoxyribonucleic acid
cRNA: Complementary Ribonucleic acid
GO: Gene Ontology
HY: High-yielding
LY: Low-yielding
qPCR: Quantitative Real Time Polymerase Chain Reaction
RNA: Ribonucleic acid
TCA: Tricarboxylic Acid
WAP: Week after pollination
