# Supplementary material for: Differential gene expression at different stages of mesocarp development in high- and low-yielding oil palm
Source: BMC Genomics. 2017 Jun 21;18:470. doi: 10.1186/s12864-017-3855-7 (PMC5480177; doi:10.1186/s12864-017-3855-7)
Supplement: Supplementary file 2 — qPCR primer efficiencies and gene expression comparison between HY and LY oil palm group at specific time points for selected differentially expressed genes. (DOCX 66 kb) [file 12864_2017_3855_MOESM2_ESM.docx]

Supplementary data 2

QPCR Primers Efficiency

| Gene annotation | Primer efficiency (%) |
| --- | --- |
| Sucrose phosphatase 2 | 102.257 |
| KAS I | 106.331 |
| KAS III | 69.145 |
| GAPDH | 74.12 |
| ATP Citrate Lyase | 100.206 |
| MADS34 | 98.05 |
| MYB44 | 99.623 |
| Beta-fructofuranosidase 1 | 88.772 |
| Starch synthase 1 | 83.621 |
| Sucrose synthase 2 | 94.995 |
| PDH (E1 component) | 89.506 |
| MADS21 | 93.912 |
| 4-alpha-glucanotransferase | 109.36 |

Selected Gene Expression comparison between HY and LY oil palm group at specific time point (WAP) Using QPCR

Green: High Oil Yielding Group (HY), Red: Low Oil Yielding Group (LY)

cDNA used in qPCR study was pooled of biological samples of HY and LY at every time point. The error bar represented the standard deviation of mean in 3 technical replicates of qPCR reaction.

Green: High Oil Yielding Group (HY), Red: Low Oil Yielding Group (LY)

cDNA used in qPCR study was pooled of biological samples of HY and LY at every time point. The error bar represented the standard deviation of mean in 3 technical replicates of qPCR reaction.

Green: High Oil Yielding Group (HY), Red: Low Oil Yielding Group (LY)

cDNA used in qPCR study was pooled of biological samples of HY and LY at every time point. The error bar represented the standard deviation of mean in 3 technical replicates of qPCR reaction.
