# Supplementary material for: Differential gene expression at different stages of mesocarp development in high- and low-yielding oil palm
Source: BMC Genomics. 2017 Jun 21;18:470. doi: 10.1186/s12864-017-3855-7 (PMC5480177; doi:10.1186/s12864-017-3855-7)
Supplement: Supplementary file 4 — Expression patterns of ACCase Subunit Alpha and genes involved in starch and sucrose metabolism pathway throughout fruit ripening in oil palm (12 – 22 WAP). (DOCX 1353 kb) [file 12864_2017_3855_MOESM4_ESM.docx]

Supplementary data 4

Differentially expression pattern of ACCase Subunit Alpha throughout fruit ripening in oil palm (12 – 22 WAP) in microarray.


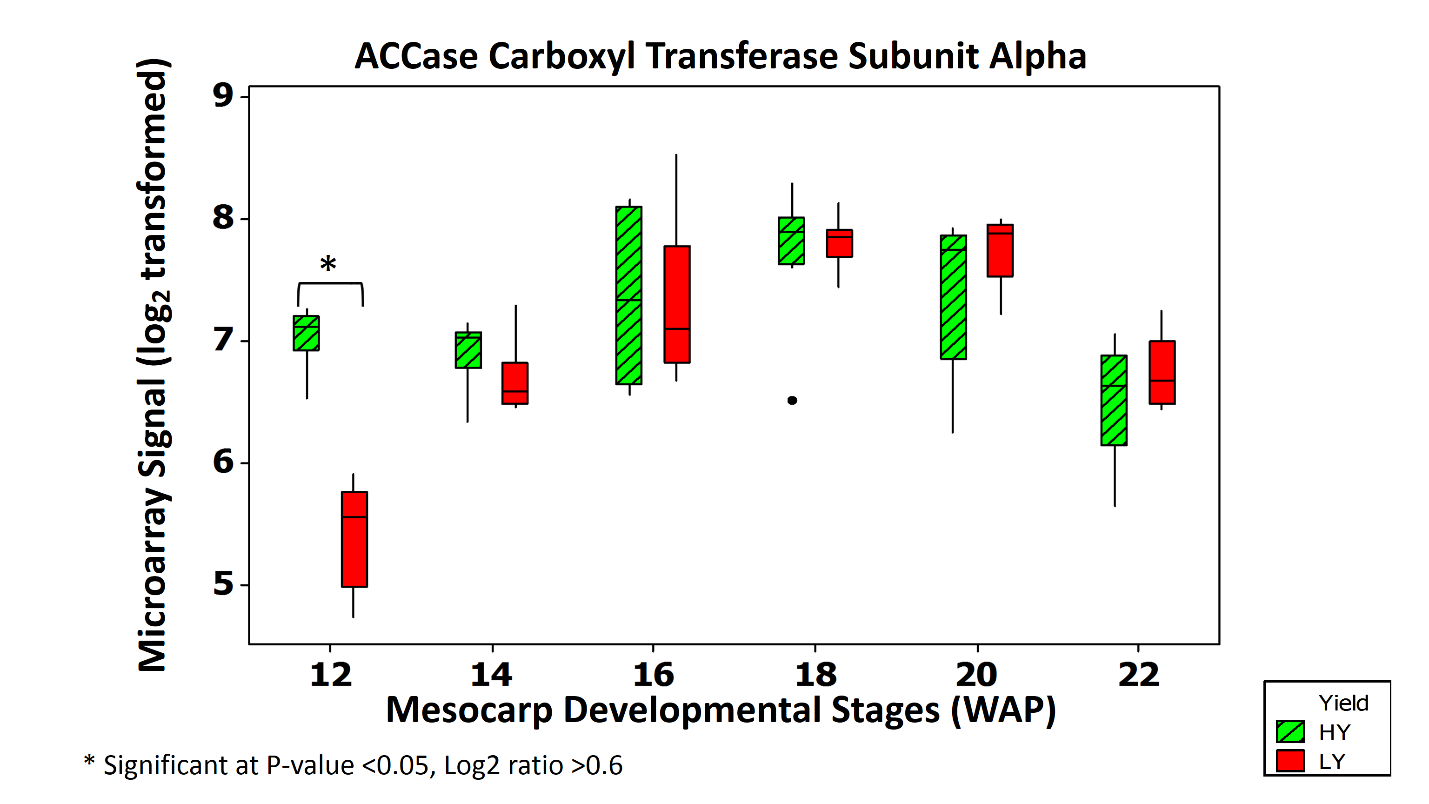


* Significant differentially expressed time point between HY and LY oil palms, p-value < 0.05, Log2 fold change ≥ 0.6 for microarray. The error represent the standard deviation of the mean for 8 biological samples for 12-20 WAP and 6 HY; 7 LY for 22 WAP.

Differentially expressed genes involved in starch and sucrose metabolism pathway.

(A)


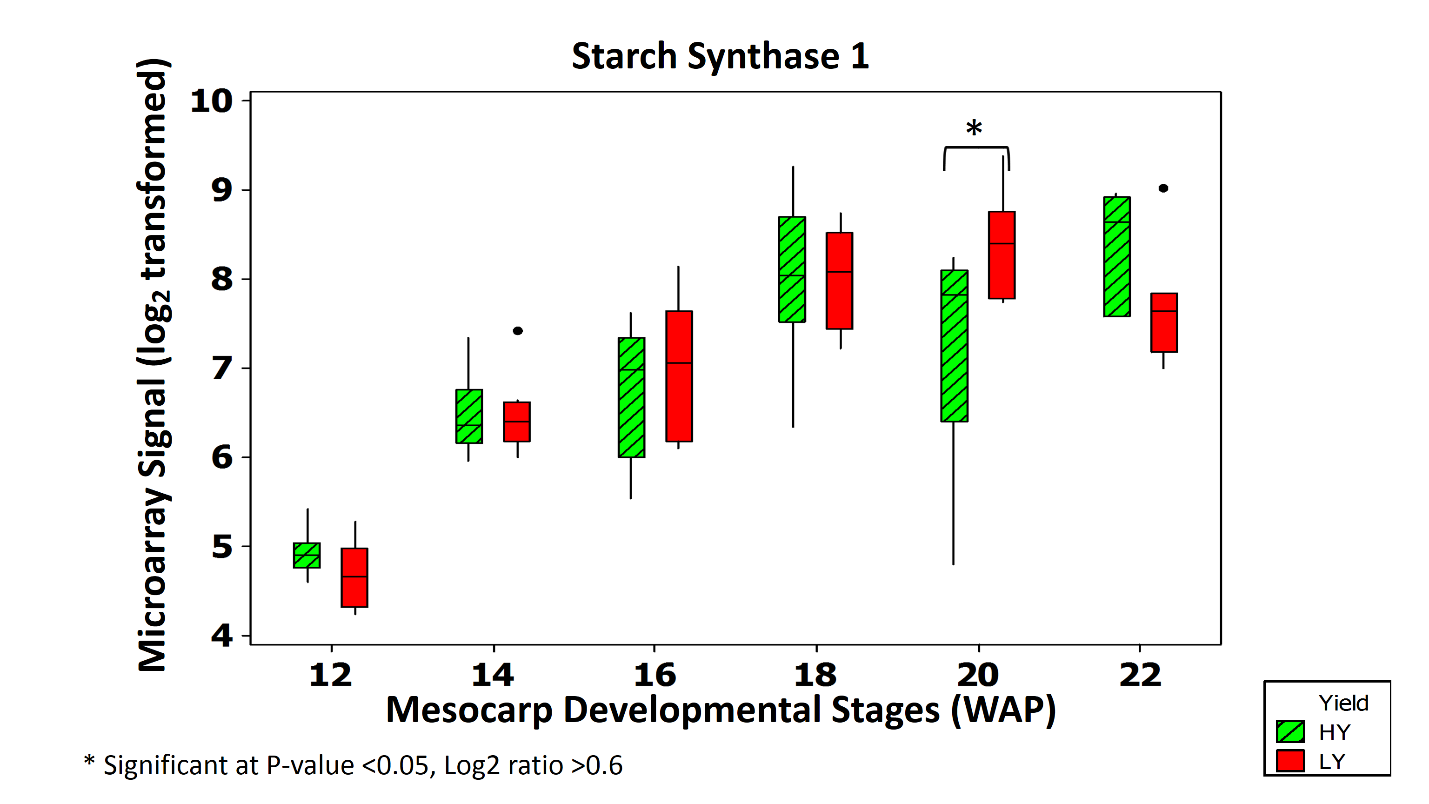


(B)


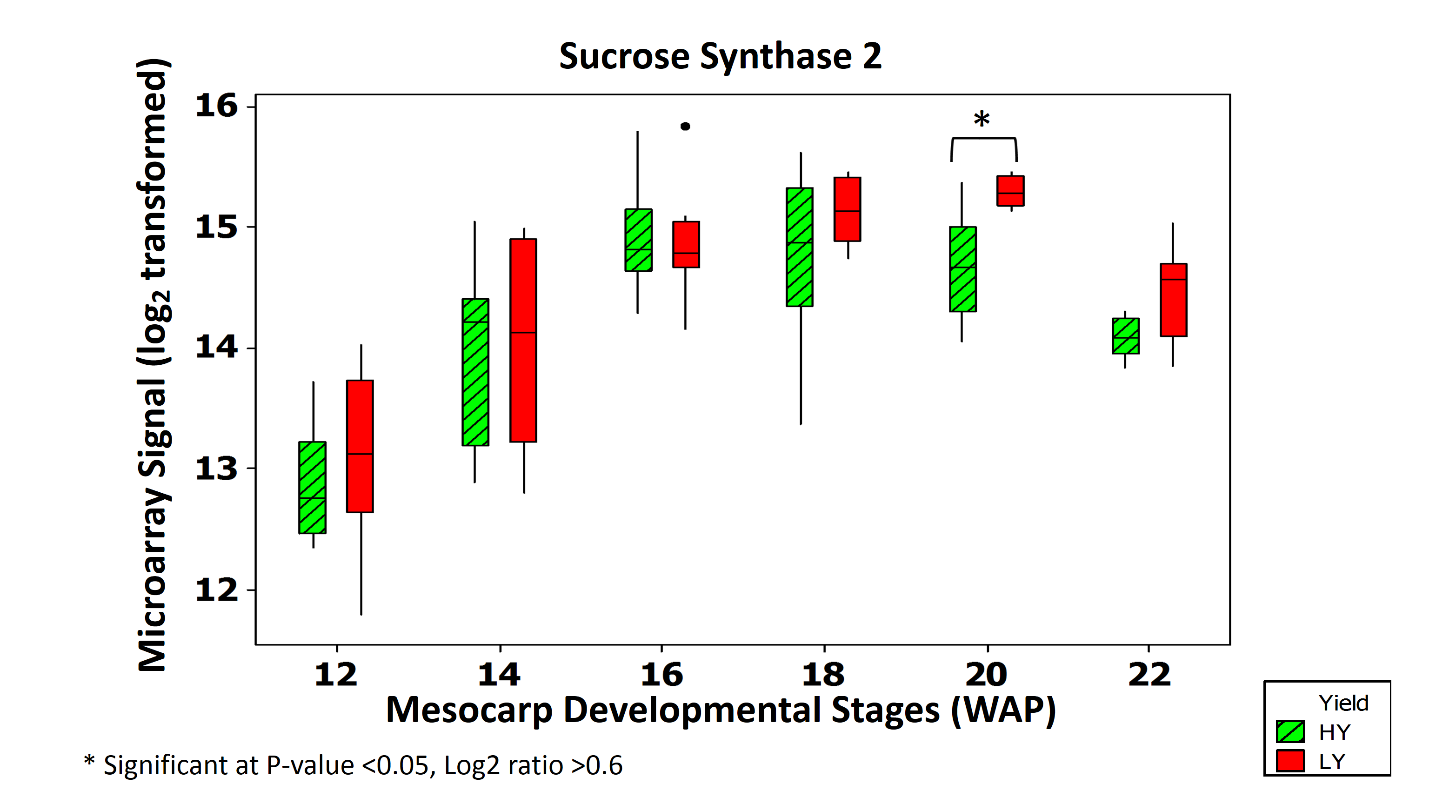


(C)


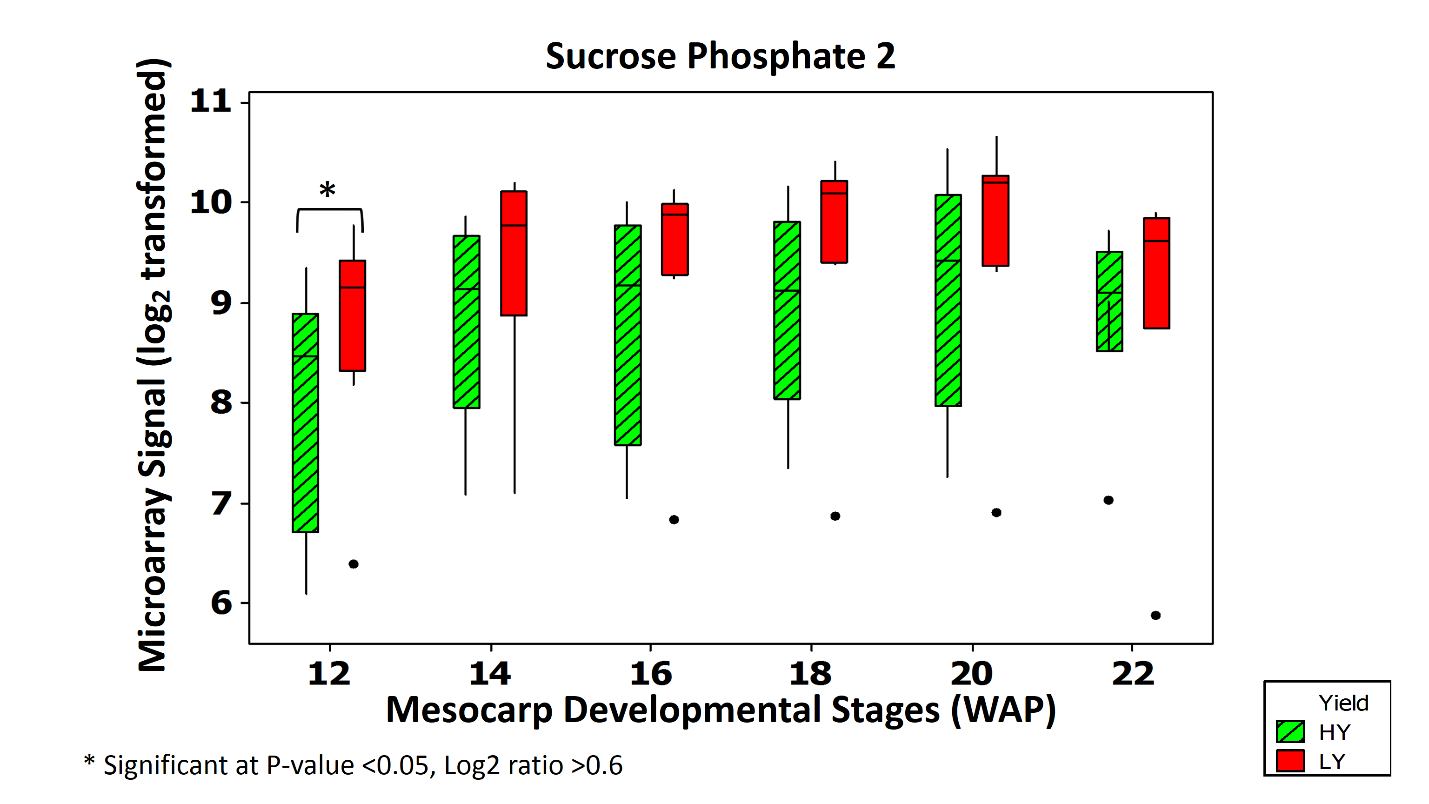


(D)


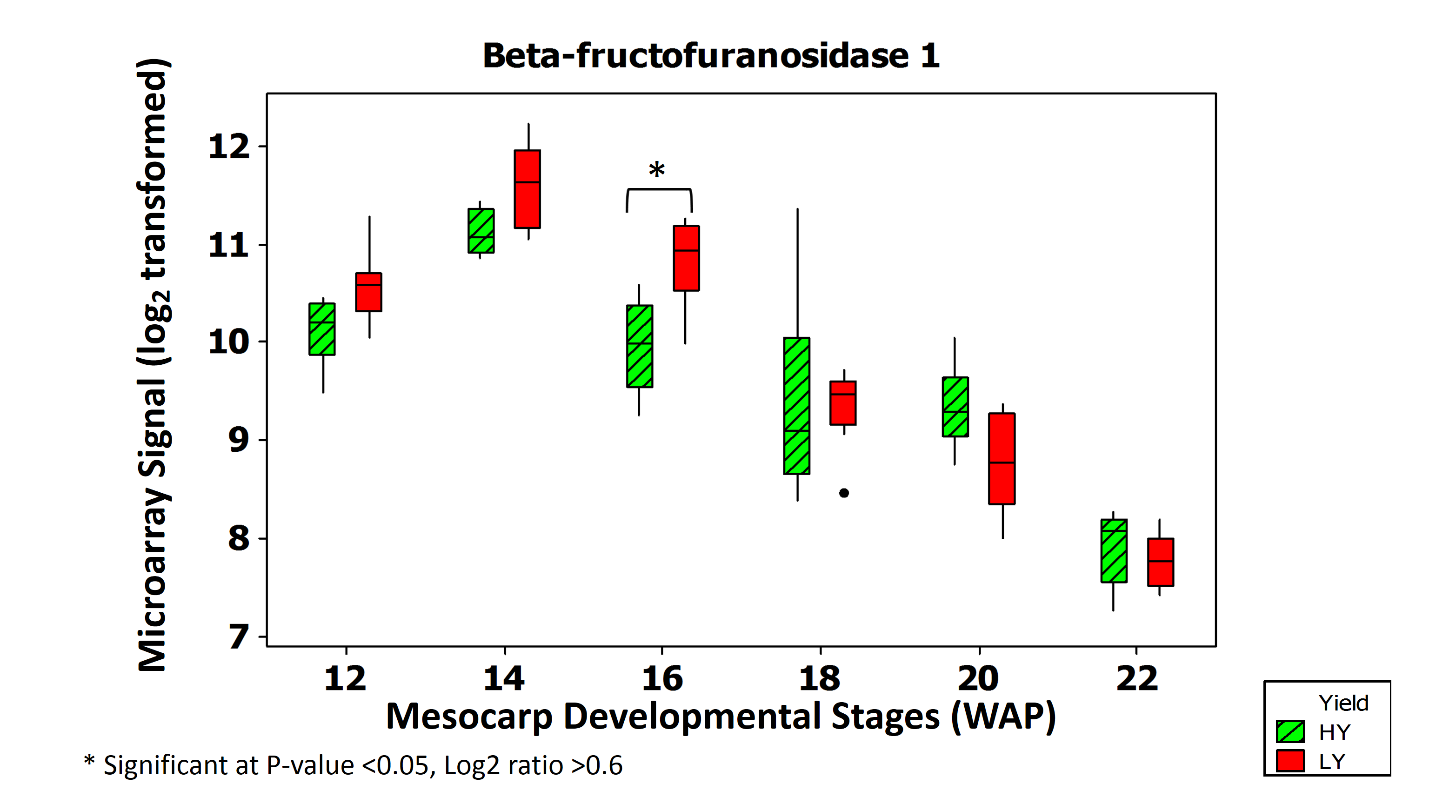


(E)


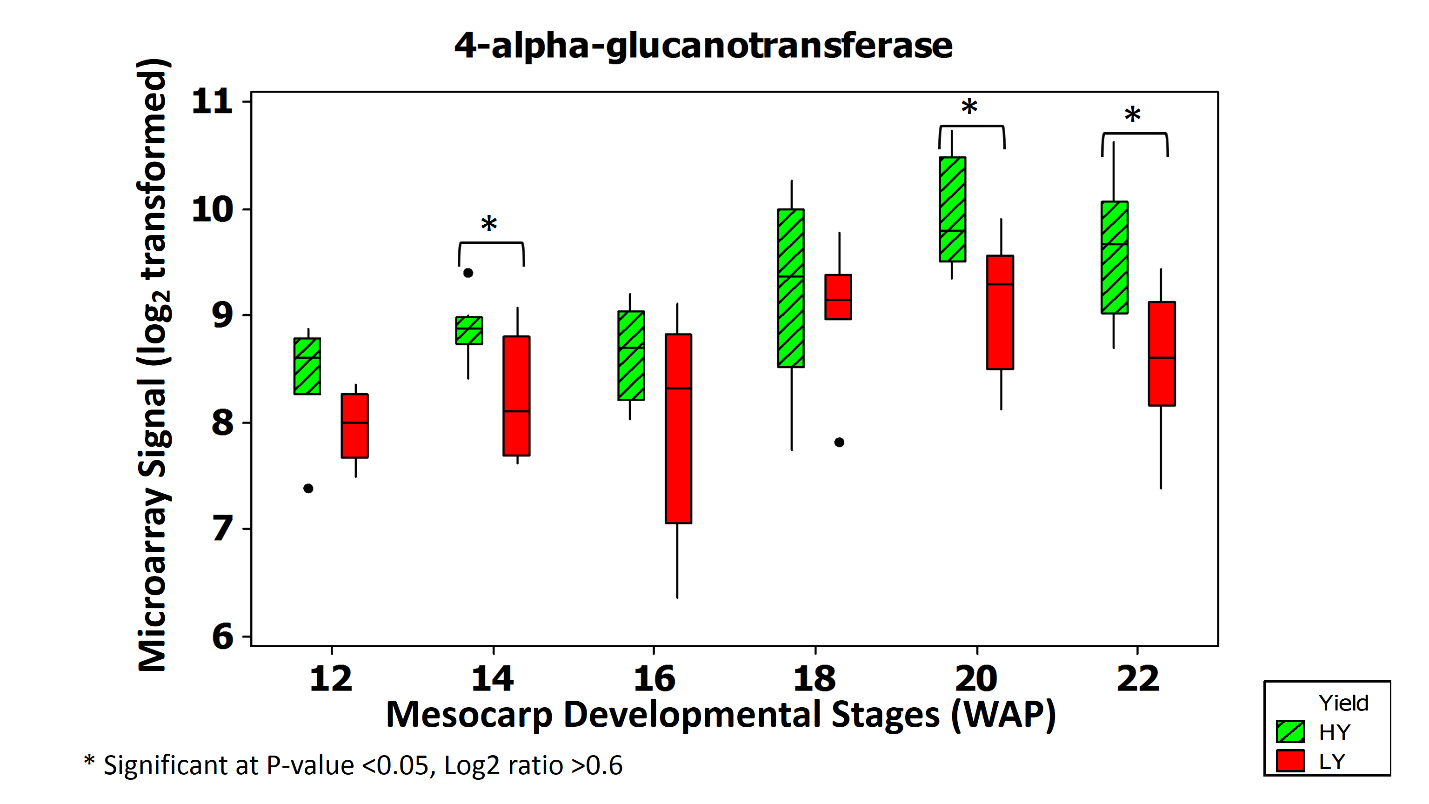


* Significant differentially expressed time point between HY and LY oil palms, p-value < 0.05, Log2 fold change ≥ 0.6. The error represent the standard deviation of the mean for 8 biological samples for 12-20 WAP and 6 HY; 7 LY for 22 WAP.
